# India-Rubber Ring on a Mackerel

**Published:** 1882-02

**Authors:** 


### INDIA-RUBBER RING ON A MACKEREL.

A rather singular incident in connection with the life of a mackerel came to hand here yesterday. While one of our small mackerel boats was fishing for mackerel with hook and line in our bay a mackerel was caught with an india-rubber band around it, and which had been there for a long time as the skin under the band showed considerable abrasion, with here and there occasional wounds. The probabilities are that about a year ago a fishing pleasure party were out somewhere along our coast who had been drinking ginger beer, when small mackerel of six or seven inches were probably plentiful, and one of the young, restless imps who are always to be found among such a party, must have drawn out the elastic ring or band from the bottle and slipped it over the head of a live mackerel, and just below the pectoral fins, and then have thrown the fish back again into the sea; and, as a consequence, the poor mackerel must have had a weary and miserable year of it. And here, I am sure, some of our lady social reformers may learn a lesson on tight lacing with a vengeance, for, as the fish fed and increased in size, the band kept its firm grip around him and only allowed him to grow to the extent of the elasticity of the band; and so tenacious was the hold, that whereas the mackerel had grown to eleven inches in length and four inches in circumference above and below the band, under it the girth was only three inches. In fact, the poor mackerel had really a wasp waist. Moreover, from this ligature being so constantly and firmly around the fish I estimate that it lost from its natural growth two inches in length and one inch in girth in the year.—*Cornwall (Eng.) Correspondence in Land and Water.*

